# iPS Cell Cultures from a Gerstmann-Sträussler-Scheinker Patient with the *Y218N PRNP* Mutation Recapitulate tau Pathology

**DOI:** 10.1007/s12035-017-0506-6

**Published:** 2017-05-02

**Authors:** Andreu Matamoros-Angles, Lucía Mayela Gayosso, Yvonne Richaud-Patin, Angelique di Domenico, Cristina Vergara, Arnau Hervera, Amaya Sousa, Natalia Fernández-Borges, Antonella Consiglio, Rosalina Gavín, Rakel López de Maturana, Isidro Ferrer, Adolfo López de Munain, Ángel Raya, Joaquín Castilla, Rosario Sánchez-Pernaute, José Antonio del Río

**Affiliations:** 1 Institute for Bioengineering of Catalonia (IBEC), Parc Científic de Barcelona, Baldiri Reixac 15-21, E-08028 Barcelona, Spain; 2 Department of Cell Biology, Physiology and Immunology, Universitat de Barcelona, Barcelona, Spain; 3 Centro de Investigación Biomédica en Red sobre Enfermedades Neurodegenerativas (CIBERNED), Barcelona, Spain; 4 Institute of Neuroscience, University of Barcelona, Barcelona, Spain; 5 Stem cells and neural repair laboratory, Fundación Inbiomed, San Sebastian, Gipuzkoa Spain; 6 Proteomics unit (Prion lab), CIC bioGUNE, Parque tecnológico de Bizkaia, 48160 Derio, Bizkaia Spain; 7 IKERBASQUE, Basque Foundation for Science, Bilbao, Bizkaia Spain; 8 Centre de Medicina Regenerativa de Barcelona, c/ Dr. Aiguader 88, 08003 Barcelona, Spain; 9 Centro de Investigación Biomédica en Red en Bioingeniería, Biomateriales y Nanomedicina (CIBERBBN), Madrid, Spain; 10 Institut de Biomedicina de la Universitat de Barcelona, Barcelona, Spain; 11 Dept. Patologia i Terapèutica Experimental, Universitat de Barcelona, Barcelona, Spain; 12 Present Address: Laboratory of Histology, Neuroanatomy and Neuropathology (CP 620), ULB Neuroscience Institute. Université Libre de Bruxelles, Faculty of Medicine, Brussels, Belgium; 13 Present Address: CISA-INIA, Center for Animal Health Research, Madrid, Spain; 14 Department of Molecular and Translational Medicine, University of Brescia, Brescia, Italy; 15 Instituto Biodonostia-Hospital Universitario Donostia, San Sebastian, Gipuzkoa Spain; 16 Neurosciences Department, University of the Basque Country UPV-EHU, Bilbao, Spain; 17 Centro de Investigación Biomédica en Red sobre Enfermedades Neurodegenerativas (CIBERNED), San Sebastian, Gipuzkoa Spain; 18 Institució Catalana de Recerca i Estudis Avançats (ICREA), Barcelona, Spain; 19 Present Address: Andalusian Initiative for Advanced Therapies, Junta de Andalusia, Seville, Spain

**Keywords:** Gerstmann-Sträussler-Scheinker, Induced pluripotent stem cells, Tau, Cellular prion protein

## Abstract

Gerstmann-Sträussler-Scheinker (GSS) syndrome is a fatal autosomal dominant neurodegenerative prionopathy clinically characterized by ataxia, spastic paraparesis, extrapyramidal signs and dementia. In some GSS familiar cases carrying point mutations in the *PRNP* gene, patients also showed comorbid tauopathy leading to mixed pathologies. In this study we developed an induced pluripotent stem (iPS) cell model derived from fibroblasts of a GSS patient harboring the *Y218N PRNP* mutation, as well as an age-matched healthy control. This particular *PRNP* mutation is unique with very few described cases. One of the cases presented neurofibrillary degeneration with relevant Tau hyperphosphorylation. *Y218N* iPS-derived cultures showed relevant astrogliosis, increased phospho-Tau, altered microtubule-associated transport and cell death. However, they failed to generate proteinase K-resistant prion. In this study we set out to test, for the first time, whether iPS cell-derived neurons could be used to investigate the appearance of disease-related phenotypes (i.e, tauopathy) identified in the GSS patient.

**Electronic supplementary material:**

The online version of this article (doi:10.1007/s12035-017-0506-6) contains supplementary material, which is available to authorized users.

## Introduction

Biomedical research on neurodegenerative diseases with low prevalence in humans relies on the possibility of analyzing brain samples only at very late stages of the disease. Thus, our view of the biochemical or molecular changes during the disease is partial. This drawback steadily increases with a faster neurodegenerative progression speed (e.g., in prionopathies [1] or rapid Alzheimer’s disease [2]). This is also the case for most sporadic taupathies and in most cases of frontotemporal lobar degeneration (FTLD) displaying neurofibrillary degeneration [3, 4]. This limitation impedes the study of early onset changes in asymptomatic patients, making it impossible to investigate illness evolution, therefore hampering biochemical/molecular studies and drug discovery [5].

Gerstmann-Sträussler-Scheinker syndrome (GSS) is a rare autosomal dominant neurodegenerative prionopathy clinically characterized by a wide spectrum of manifestations including but not limited to ataxia, spastic paraparesis, extrapyramidal signs and dementia [6, 5]. Most GSS patients have the *P102L* mutation in the cellular prion protein (PrP^C^) gene (*PRNP*) located in the short arm of chromosome 20 [5]. Cases of rapid progressive forms of GSS are rare [7] with an average duration after clinical diagnosis of 5–6 years (from 6 months to 13 years) [8, 5]. Histopathological examination of post-mortem GSS brains has revealed abnormal misfolded prion (PrP) aggregates in the form of unicentric and multicentric deposits in the cerebellum and cortical gray matter [5]. In addition, western blot analysis of aggregated PrP is distinguished by the presence of truncated protein fragments ranging between 6 and 10 KDa and a variable number of bands of higher molecular weight [9]. Parallel to this particular PrP deposition, pathological features characteristic of other neurodegenerative diseases such us parkinsonism or Alzheimer’s disease have been observed in some GSS patients [5]. Indeed, an increase in hyperphosphorylated Tau is frequently observed in the pathological analysis of brains from GSS patients carrying *PRNP* mutations *P102L* [10], *P105L* [11], *A117V* [12], *V176G* [13], *F198S* [14, 15], *Q217R* [16, 15] and *Y218N* [17]. Although it has been shown that PrP^C^ with the *P102L* mutation display an increased binding to Tau [18], the role of these point mutations in the development of neurofibrillary degeneration is unknown. Nevertheless, in some *P102L* GSS cases with increased levels of p-Tau, the distribution of p-Tau tangles close to PrP deposits suggesting an active participation of PrP in the generation of p-Tau [10].

Due to the above-mentioned restrictions in this study we explored the usefulness of an induced pluripotent stem (iPS) cell model derived from somatic cells from a GSS patient. iPS cell technology is a tool for basic and translational research through generating in vitro models of disease-relevant cells reprogrammed directly from patients [19–21]. This approach has been shown to be particularly useful in the case of congenital or early-onset monogenic diseases [22] as well as other neurodegenerative diseases [23]. iPS cells have been generated from patients with Alzheimer’s [24], Parkinson’s [25, 26], Hungtinton’s [27] diseases as well as FTLD [28], Amyotrophic Lateral Sclerosis (ALS) [29] and several others. However, there are no reports of iPS cell lines derived from patients with familial prionopathies.

In this study, we generated iPS cells from dermal fibroblasts of a family member of the *Y218N* GSS patient described by Alzualde and colleagues [17] and differentiated them into neurons using two previously published procedures [30, 31]. To date, very few individuals have been reported carrying this mutation [17, 32]. We were interested in this familiar since the *Y218N* patient displayed widespread neurofibrillary degeneration in the brain [17]. Results determined that although differentiated *Y218N* iPS cells were not able to spontaneously generate or propagate human prions, *Y218N*-derived cultures showed relevant astrogliosis and cell death. In addition, differentiated *Y218N*-derived neurons displayed high levels of p-Tau, thus recapitulating most of the neuropathological features reported in the patient [17].

## Material and Methods

### Case Patient

The index case and the younger sister was examined at the Cognitive Disorders Unit at Donostia Hospital. The clinical report of the family and the *Y218N* patient can be seen in [17]. Dermal fibroblasts were obtained from the younger sister of the *Y218N* patient (54 years old in 2010) after having made complaints of poor concentration, apathy, emotional lability, and increasing difficulties in planning and executing actions. She had previously been diagnosed with and treated for a depressive illness, and the neuropsychological examination revealed slight memory dysfunction in retrieval, language impairment followed by anomia with preserved verbal comprehension, and executive dysfunction. The Mini Mental State Examination (MMSE) score was 23/30. Magnetic resonance imaging showed slight frontotemporal atrophy and EEG analysis revealed intermittent frontotemporal delay. An additional EEG, 6 months later, showed slow background activity in the patient, with intermittent delta waves in the left hemisphere. 10 months after onset, she had language difficulties, with impairment in semantic knowledge, and MMSE score dropped to 13/30.

### Generation of iPS Cells

All experiments were performed under the guidelines and protocols of the Ethical Committee for Animal Experimentation (CEEA) of the University of Barcelona. All procedures adhered to internal and EU guidelines for research involving derivation of pluripotent cell lines. All subjects gave informed consent for the study using forms approved by the Ethical Committee on the Use of Human Subjects in Research at Hospital Donostia in San Sebastián, Spain. Generation of iPSC lines was approved by the Advisory Committee for Human Tissue and Cell Donation and Use, by the Commission on Guarantees concerning the Donation and Use of Human Tissues and Cells of the Carlos III Health Institute, Madrid, Spain (Ref: 589, 1/21/2015). All procedures were done in accordance with institutional guidelines and the cell lines have been (or will be) deposited at the Banco Nacional de Lineas Celulares (BNLC, ISCIII) following the Spanish legislation. Fibroblasts from a healthy individual and from the *Y218N* GSS patient were infected with retroviruses carrying human cDNA coding for *KLF4*, *SOX2*, and *OCT4*, with or without the addition of *c-MYC* as previously described [33]. Fibroblasts were maintained in DMEM (Sigma) supplemented with 10% FBS (Life Technologies) and 1% Pen/Strep solution (Life Technologies) before infection. After infection, fibroblasts were plated on irradiated human foreskin fibroblasts (HFF, ATCC) and maintained with hESC medium for 4–12 weeks until iPS cell colonies appeared. Several clones from each cell line were obtained and validated. *Y218N* patient (FH10) and parallel control (FHB1) iPS cell clones were analyzed in details (see below).

### Characterization of iPS Cell Lines

AP staining was performed using the Alkaline Phosphatase Blue Membrane Substrate Solution (Sigma). For immunocytochemistry, cells were grown on HFF feeder layers for 6–10 days and then fixed in 4% PFA for 10 min. After embryonic bodies (EB) formation, differentiation into the 3 germ layers was performed. For endoderm, EBs were plated on 6-well plates treated with Matrigel (BD Biosciences) for 1 h at room temperature, and maintained for 28 days with EB medium. The same procedure was used for mesoderm, but instead using EB medium with 0.5 mM of ascorbic acid. For ectoderm differentiation, EBs were maintained in suspension for 10 days with Neurobasal medium containing N2, B27 and FGF2 (N2B27 medium), prepared as previously described [26]. EBs were then plated on 6-well Matrigel-coated plates and maintained for 21 days with N2B27 medium without FGF supplementation. Differentiated cells were fixed in 4% PFA for 10 min. For nuclear DAPI staining (Invitrogen), 0.5 μg/ml was used. The slides were mounted with PVA:DABCO mounting medium. Images were acquired with an SP2 confocal system (Leica) and analyzed with ImageJ™ software. RT-qPCR analysis was performed as previously described [26]. All results were normalized to the average expression of Glyceraldehyde 3-phosphate dehydrogenase (*GAPDH*). Transcript-specific primers used are shown in Supplementary Table 1.

For karyotyping, iPS cells were grown on Matrigel and treated with colcemid (Life Technologies) at a final concentration of 20 ng/ml. Karyotyping analysis was carried out by Prenatal Genetics S.L. (Barcelona). For promoter methylation, testing reprogramming gene integration and sequencing to confirm that patient iPS cells were carrying mutations in *PRNP* gene, DNA was isolated using the QIAamp DNA Mini Kit (QIAGEN) following the manufacturer’s instructions. Bisulfit conversion of the promoters was carried out using the Methylamp DNA modification kit (Epigentek). Five clones of each promoter for each cell line were analyzed by sequencing. The primers used for testing gene integration are shown in Supplementary Table 1.

### iPS Cell Differentiation to Neural Cells

In this study, two protocols were used to differentiate the iPS cells. In the first protocol, iPS cell colonies were mechanically passaged onto Matrigel-coated 6-well plates. 24 h later the mTeSR™ was replaced by DDM neural induction medium [34, 35] with the addition of the ALK inhibitor, SB431542 at 10 mM for 4 days (Tocris) and the BMP inhibitor, LDN-193189 at 100 nM (Miltenyi Biotech) for 12 days. Cells were propagated in this medium for 3 weeks. At about 24 days in vitro, cells were dissociated and plated onto wells coated with poly-L-lysine (33.3 mg/ml, BD) and laminin (3.3 mg/ml, BD), and the medium was changed to N2B27 medium. For immunofluorescence, neurons were dissociated with Accutase (Sigma) and replated on glass coverslips coated with poly-L-lysine and laminin. Characterization was done as previously described [36].

In the second procedure, spherical neural masses (SNMs) were obtained as previously described [37]. SNMs were fixed in 4% phosphate buffered paraformaldehyde (PFA) for 2 h and characterized by immunostaining. For nuclear DAPI staining (Invitrogen), 5 μg/ml was used. Mounting medium and imaging analysis were performed for in vitro differentiation testing. SNMs obtained from control and *Y218N* iPS cells, having been maintained in suspension, were then plated on slide-flasks, 6-well plates, 35 mm ∅ plates or 10 mm ∅ plates all previously treated with Matrigel for 1 h at room temperature, and differentiated for 3, 6 or 9 weeks with N2B27 [26], without FGF supplementation to obtain neural cultures. The correct differentiation was assessed by immunostaining. Antibodies used are shown in Supplementary Table 2. For nuclear DAPI staining (Invitrogen), 0.5 μg/ml was used. The slides were mounted with Mowiol mounting medium.

### RT-PCR Protocol

Quantitative real time PCR was performed on total RNA extracted with mirVana’s isolation kit (Ambion) from differentiating iPS cells. Purified RNAs were used to generate the corresponding cDNAs, which served as PCR templates for mRNA quantification. Quantitative RT-PCR assays were performed in duplicate on cDNA samples obtained from the retro-transcription reaction diluted 1:20 in 384-well optical plates (Kisker Biotech) using the ABI Prism 7900 HT Sequence Detection System (Applied Biosystems). The reactions were carried out using 20xTaqMan gene expression assays for genes and 2xTaqMan Universal PCR Master Mix (Applied Biosystems). The reactions were conducted using the following parameters: 50 °C for 2 min, 95 °C for 10 min, 40 cycles at 95 °C for 15 s and 60 °C for 1 min. The fold change was determined using the eq. 2^-ΔΔCT^. Primers used in iPS cell differentiation experiments and Tau R3/R4 analysis can be seen in Supplementary Table 3.

### Sample Collection and Proteinase K Treatment

Samples of control and *Y218N* differentiating cultures were collected at several differentiation times and were homogenized in 10% lysis buffer (100 mM NaCl, 10 mM EDTA, 0.5% Nonidet P-40, 0.5% sodium deoxycholate, and 10 mM Tris, pH 7.5). Debris were removed with low-speed centrifugation at 3000×*g* for 10 min, and the supernatants were collected. To detect the presence of Proteinase K (PK)-resistant PrP in the supernatant, homogenates were digested with a final concentration of 10–50 μg/ml PK at 37 °C for 60 min prior to western blot analysis using 3F4 antibody against PrP^C^. To evaluate the PK resistance of protein samples from the original *Y218N* patient [17], type I sporadic Creutzfeldt-Jakob disease (sCJD) and Type II sCJD brain homogenates were also processed in parallel. PK digestion was terminated by adding Laemmli buffer and heating the samples at 100 °C for 10 min.

### Western Immunoblot

Samples from different differentiation stages from iPS cells to neuronal cultures were processed for western blot, including human post-mortem samples and control cultured cells. The collected samples were homogenized in (10% wt/vol) of 50 mM Tris-HCl, pH 7.4/150 mM NaCl/0.5% Triton X-100/0.5% Nonidet P-40 and a mixture of proteinase inhibitors. After this, samples were centrifuged at 15,000 x *g* for 20 min at 4 °C. The resulting supernatant was normalized for protein content using BCA kit (Pierce). Cell extracts containing Laemmli buffer were boiled at 100 °C for 10 min, followed by 8–10% SDS electrophoresis, then electrotransferred to nitrocellulose membranes for 2 h at 4 °C. Membranes were then blocked with 5% not-fat milk in 0.1 M Tris-buffered saline (pH. 7.4) for 2 h and incubated overnight in 0.5% blocking solution containing primary antibodies. After incubation with peroxidase-tagged secondary antibodies (1:2000 diluted), membranes were revealed with ECL-plus chemiluminescence western blot kit (Amershan-Pharmacia Biotech). In our experiments, each nitrocellulose membrane was used to detect p-Tau (AT-8 and PHF1 antibodies), Actin, Tubulin as protein loading controls. A list of the antibodies used in these experiments can be seen in Supplementary Table 2.

### Densitometry and Statistical Processing

For quantification, developed films were scanned at 2400 × 2400 dpi (i800 MICROTEK high quality film scanner), and the densitometric analysis was performed using Quantity One Image Software Analysis (Biorad). Statistical analysis of the obtained data (RT-qPCR and Western blot) was performed using Bonferroni post hoc test (Multiple comparison test) using GraphPad Prism 6 (Mac OsX, Grahpad). Data are presented as mean ± standard error of the mean (S.E.M.). Differences between groups were considered statistically significant between **** *P* < 0.001, *** *P* < 0.01 and ** *P* < 0.05.

### Immunohistochemistry

Differentiating iPS cell cultures were fixed in 4% PFA at different days in culture and then permeabilized with 0.1% Triton X-100 (Sigma) in 0.1 M PBS. After fixation, and extensive rinsing with 0.1 M PBS, cultures were blocked with 10% FBS in 0.1 M PBS prior to incubation with primary antibodies (see Supplementary Table 2). After incubation with primary antibodies, cells were incubated with the pertinent Alexa Fluor-tagged secondary antibodies (Alexa-488 goat anti-mouse or Alexa-568 goat anti-rabbit) (Invitrogen-Life Technologies). Finally, cells were stained with 0.1 μM DAPI (Sigma) diluted in 0.1 M PBS, mounted on Mowiol, and viewed using an Olympus BX61 fluorescence microscope, Zeiss LSM or a Leica SP5 confocal microscopy.

### Corrected Total Cell Fluorescence (CTCF) Measurement

CTCF levels of p-Tau (red channel) and MAP2 (green channel) were measured in 150 (*Y218N*) and 165 (control) identified neurons after 21 days of differentiation using ImageJ™ software following published instructions http://sciencetechblog.com/2011/05/24/measuring-cell-fluorescence-using-imagej/. See also [38] for details. CTCF values were determined using the following formula. CTCF = Integrated Density – (Area of selected cell x Mean fluorescence of background readings). Statistical analysis of the obtained data was performed using Mann-Whitney U test using GraphPad Prism 6 (Mac OsX, Grahpad). Differences between groups were considered statistically significant between **** *P* < 0.001.

### Mitochondrial Movement Analysis in iPS Cell-Derived Neurons

SNM-derived neurons were incubated after 21 days of differentiation with MitoTracker (Molecular Probes) and filmed using a Leica TCS SP2 confocal microscope (Leica) equipped with a 63× immersion oil objective. Time-lapse series of image stacks composed of 10 images (512 × 512 pixels) were taken every 3 s over 10 min. Movies were generated at 10 frames per second. Forty-two axons were registered and analyzed in each group recorded. In all cases, a mitochondrion was considered motile when it moved more than 0.5 μm during 1 min of recording. Distances and speeds of retrograde and anterograde transport were measured, and no tracking pluging was used. ImageJ™ software was used to quantify mitochondrial movement. For each mitochondrion movement, the minimum displacement and the average over time were plotted. Statistical analysis of the obtained data was performed using Mann-Whitney U test using GraphPad Prism 6 (Mac OsX, Grahpad). Differences between groups were considered statistically significant between *** *P* < 0.01 and ** *P* < 0.05.

### Infectivity Assay

Brain homogenates (10% in sterile PBS) were made fresh the day of the infection. One aliquot was kept frozen at −80 °C to repeat the exposure 72 h after the first infection, as described [39]. Representative samples were taken to confirm the presence of PK-resistant PrP in the homogenates, following digestion with 50 μg/ml for scrapie (263 K) and Creutzfeldt-Jakob disease (CJD) brains and 12.5 μg/ml for the *Y218N* brain [40]. Control and *Y218N* forebrain neuronal cultures were infected at early (30–40) and middle (60–80) differentiation times. Neurons were replated 5–6 days before the experiment. The culture supernatant was replaced by fresh media containing 10% brain homogenate (day 1) and this was repeated 72 h later (day 3). Two days later, fresh medium (without inocula) was added without removing the supernatant. At day 10 post-inocula (dpi) the entire medium was replaced and the cells were washed several times with sterile PBS before adding fresh Neurobasal containing B27 and N2 supplements. Medium was replaced every other day for the first 2 weeks and then twice a week until cells were collected or fixed for analysis, ~2 months later. All experiments were performed in a Biosafety level 3 security laboratory.

## Results

### Generation and Characterization of *Y218N* GSS Patient-Specific iPS Cells

**Fig. 1:**
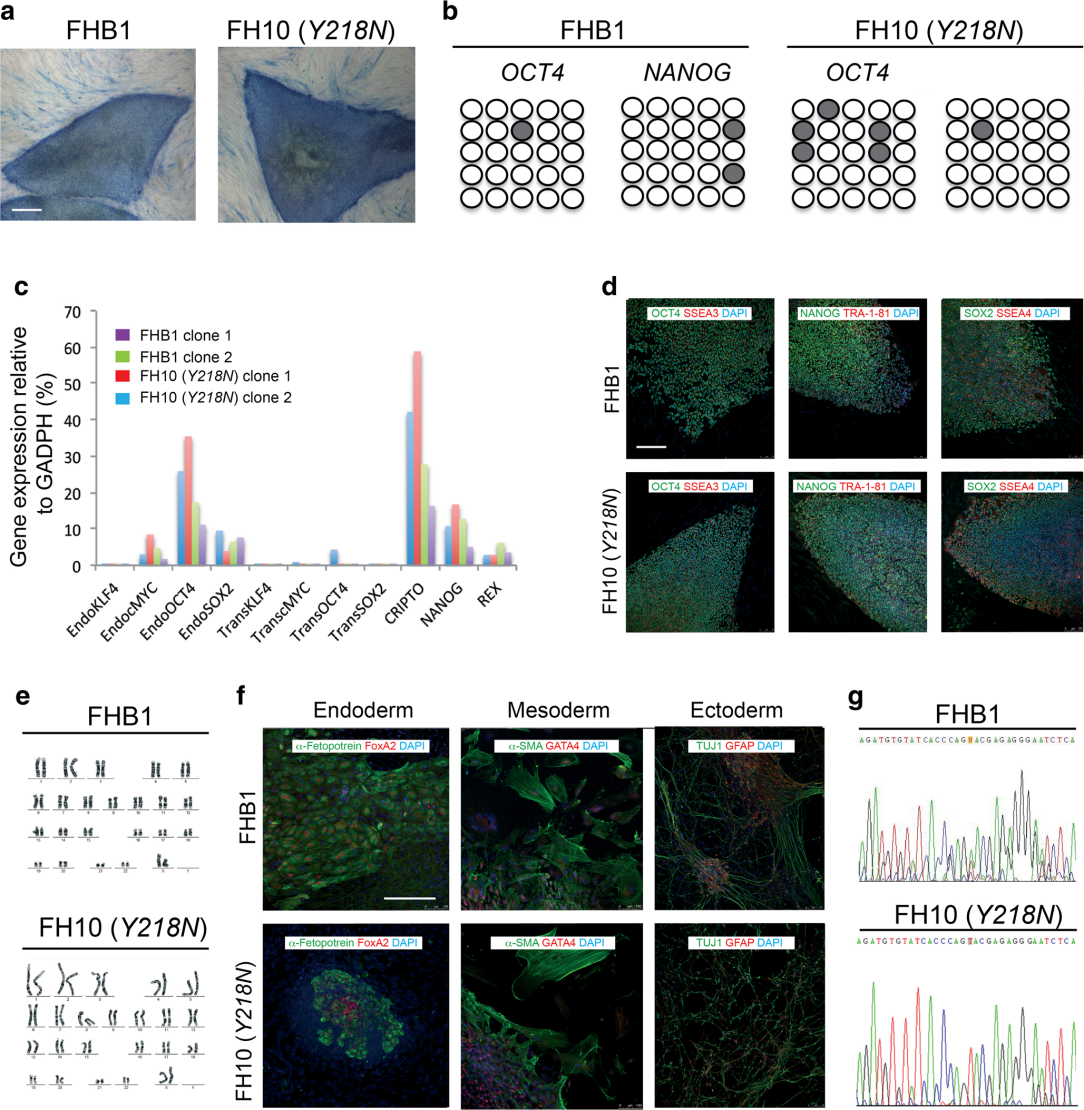
**Generation and characterization of iPS cells. (a)** Control (cell line FHB1) and GSS-*Y218N*-iPS cell (cell line FH10) stained for AP activity. (**b)** Bisulphite genomic sequencing of the *OCT4* and *NANOG* promoters showing demethylation in FHB1 and FH10 (*Y218N*) cell lines. **(c)** RT-qPCR analyses of the expression levels of retroviral-derived reprogramming factors (transgenic) and endogenous expression levels (endogenous) of the indicated genes in FHB1 (two clones) and *Y218N*-iPS cells (cell line FH10, 2 clones). **(d)** Low fluorescence photomicrographs of representative colonies of FHB1 and FH10 (*Y218N*) stained positive for the pluripotency-associated markers OCT4, NANOG and SOX2 (green), SSEA3, TRA-1-81 and SSEA4 (red). **(e)** Normal karyotypes of FHB1 and FH10 (*Y218N*) at passage 20. **(f)** Immunofluorescence analyses of FHB1 and FH10 (*Y218N*) iPS cells differentiated in vitro show the potential to generate cell derivatives of all three primary germ cell layers including ectoderm (stained for TUJ1, green), endoderm (stained for α-fetoprotein, green, and FOXA2, red) and mesoderm (stained for smooth muscle actin, SMA, red). **(g)** Direct sequence of genomic DNA from Control (cell line FHB1) and GSS patient (FH10 (*Y218N*)) identifying the *PRNP* ^*Y218N*^ mutation. Scale bars in a, d and f = 50 μm

Fibroblasts were reprogrammed at early passages (5–7) through the retroviral delivery of *SOX2*, *KLF4*, *OCT4*, and c-*MYC* to generate up to 5 independent iPS cell lines for each individual (Fig. 1). We selected clones displaying embryonic stem cell-like morphology and positive AP staining (Fig. 1a). 5 clones representing each individual were chosen to be thoroughly characterized and shown to be fully reprogrammed, as judged by demethylation of *OCT4* and *NANOG* promoters (Fig. 1b), the silencing of the reprogramming transgenes (Fig. 1c), activation of endogenous pluripotency-associated factors (Fig. 1c), expression of pluripotency-associated transcription factors and surface markers (Fig. 1d), pluripotent differentiation ability in vitro and/or in vivo (Fig. 1f), and karyotype stability after more than 15 passages (Fig. 1e). Mutation analysis confirmed that iPS cells and their derivatives bore the mutation *Y218N* present in the patient fibroblasts (Fig. 1g).

### Late Neuronal Maturation, Increased Reactive Astrogliosis and Absence of PrP Generation in *Y218N*-Derived iPS Cell Cultures

**Fig. 2:**
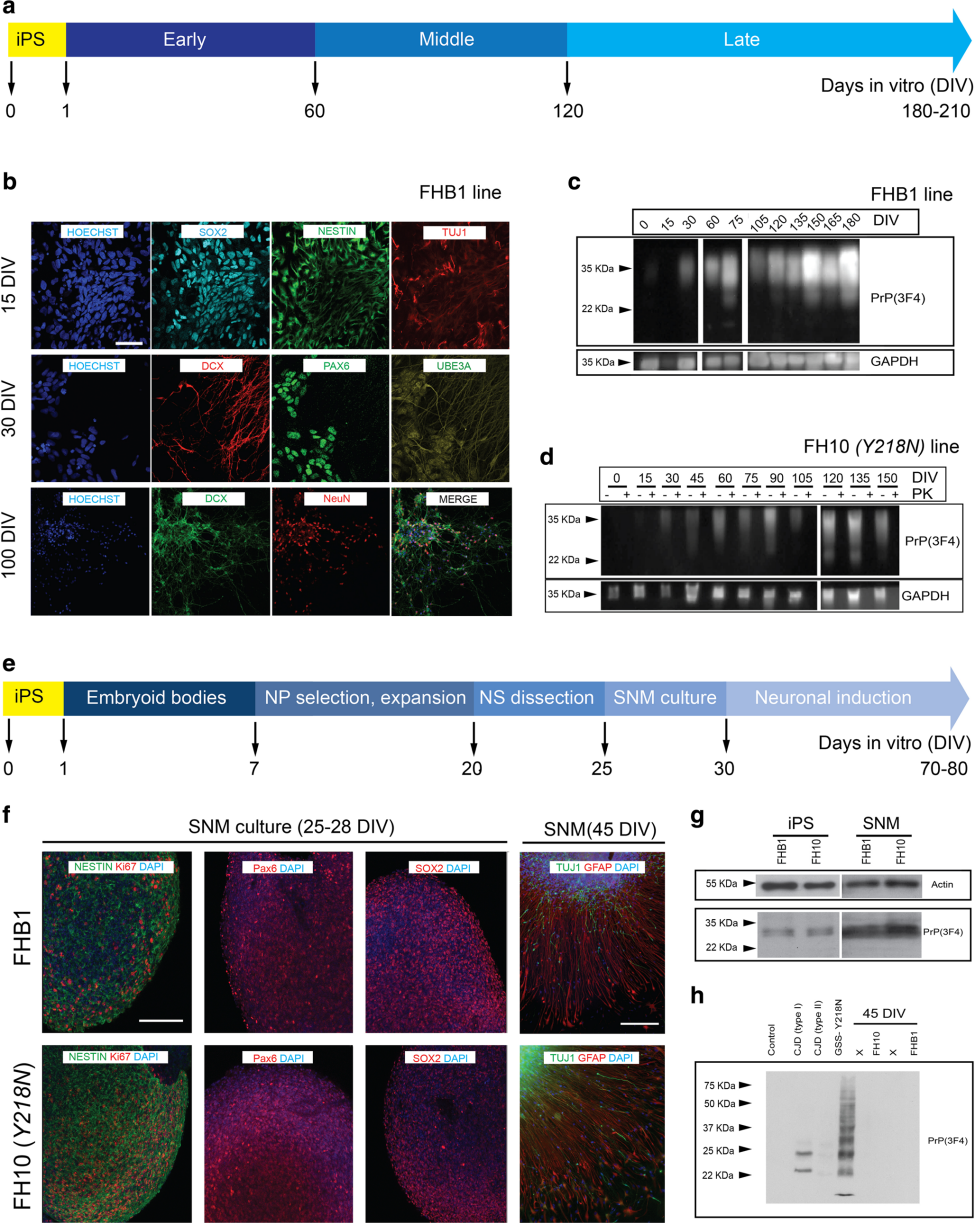
**Neural differentiation of FHB1 and FH10 (** ***Y218N*** **) iPS cells**. IPS cells from control (FHB1) and *Y218N* (FH10) GSS patient were differentiated using two procedures (**a** and **e**) (see Methods for details). **(b)** Low power photomicrographs of representative colonies of FHB1 and FH10 (*Y218N*) stained positive for SOX2, Nestin, DCX, TUJ1, PAX6, UBE3A antigens at different stages of maturation. **(c)** Western blot characterization of PrP^C^ expression in differentiating iPS cell cultures. **(d)** Example of the Western blot experiments illustrating the absence of PK-resistant PrP^C^ in FH10 (*Y218N*) cultures. **(e)** Low power photomicrographs of representative colonies of FHB1 and FH10 (*Y218N*) stained positive for Nestin, Ki67, SOX2, TUJ1, PAX6 and GFAP antigens. **(g)** Western blot characterization of PrP^C^ expression in iPS cells (passage 20) and SNMs (passage 3). **(h)** Western blots illustrating the absence of PK-resistant prion in FHB1 and FH10 (*Y218N*) in brain extracts from the GSS patient and two CJD (Type I and II) samples. Scale bars in b and f = 50 μm

Control and *Y218N*-derived iPS cells were differentiated into neural cells using two well-characterized procedures (see Methods). Neural induction was fast and efficient using both protocols (Fig. 2) and the cells sequentially expressed typical markers of neural progenitors, neuroblasts and mature neurons (Fig. 2b, f). In our first approach (Fig. 2a-d), taking into account morphology and marker expression, we established three differentiation stages: early (≈ 60 DIV), middle (≈ 60–120 DIV) and late (≈ 120–210 DIV) (Fig. 2a). Two weeks after neural induction, cultures were composed mainly of neural progenitors co-expressing SOX2 and NESTIN with a few differentiating neuroblasts (class III β-tubulin (TUJ1)-positive). By 4 weeks many neuroblasts and young neurons expressed PAX6 and the vast majority expressed Doublecortin (DCX) and Ubiquitin-protein ligase E3A (*UBE3A*) proteins (Fig. 2b). From the third month onwards, differentiating neurons expressed the mature post-mitotic neuronal marker NeuN (*RBFOX3*). In parallel and as also reported in vivo [41], PrP^C^ expression increased progressively over time during differentiation (Fig. 2c–d).

In the second protocol (Fig. 2e-h), control and *Y218N*-iPS cells were also differentiated to pure masses of neural precursors using a previously described protocol that involves the formation of EBs and the culture of neural precursor cells to form SNMs, whom can be expanded and differentiated into mature neurons after several weeks using neuronal induction medium (Fig. 2e). In these conditions, SNMs derived from control and *Y218N*-iPS cell lines homogeneously expressed neural progenitor markers such as PAX6, NESTIN, and SOX2, as well as the proliferation marker Ki67 (Fig. 2f). Furthermore, when iPS cell-derived SNMs were cultured in neuronal induction medium supplemented with N2 and B27, differentiation into mature neurons was evident within 3 to 5 weeks (Fig. 2f). After about 3 weeks in neuronal medium, the cultures formed dense MAP2 and TUJ1-positive neuronal networks (Fig. 2f) in presence of astroglial cells (not shown). No mixed genotypes (GFAP + MAP2 or TUJ1 double labeled cells) were observed. As observed in the first approach, PrP^C^ was clearly present throughout neural differentiation (Fig. 2c,g, Supplementary Fig. 1). However, no detectable PK-resistant PrP was observed in protein extracts treated with the enzyme in the *Y218N* and control-derived neurons generated with either protocol (Fig. 2d,h), in contrast to brain extracts from *Y218N* GSS or Type 1–2 CJD patients (Fig. 2h, Supplementary Fig. 2).

**Fig. 3:**
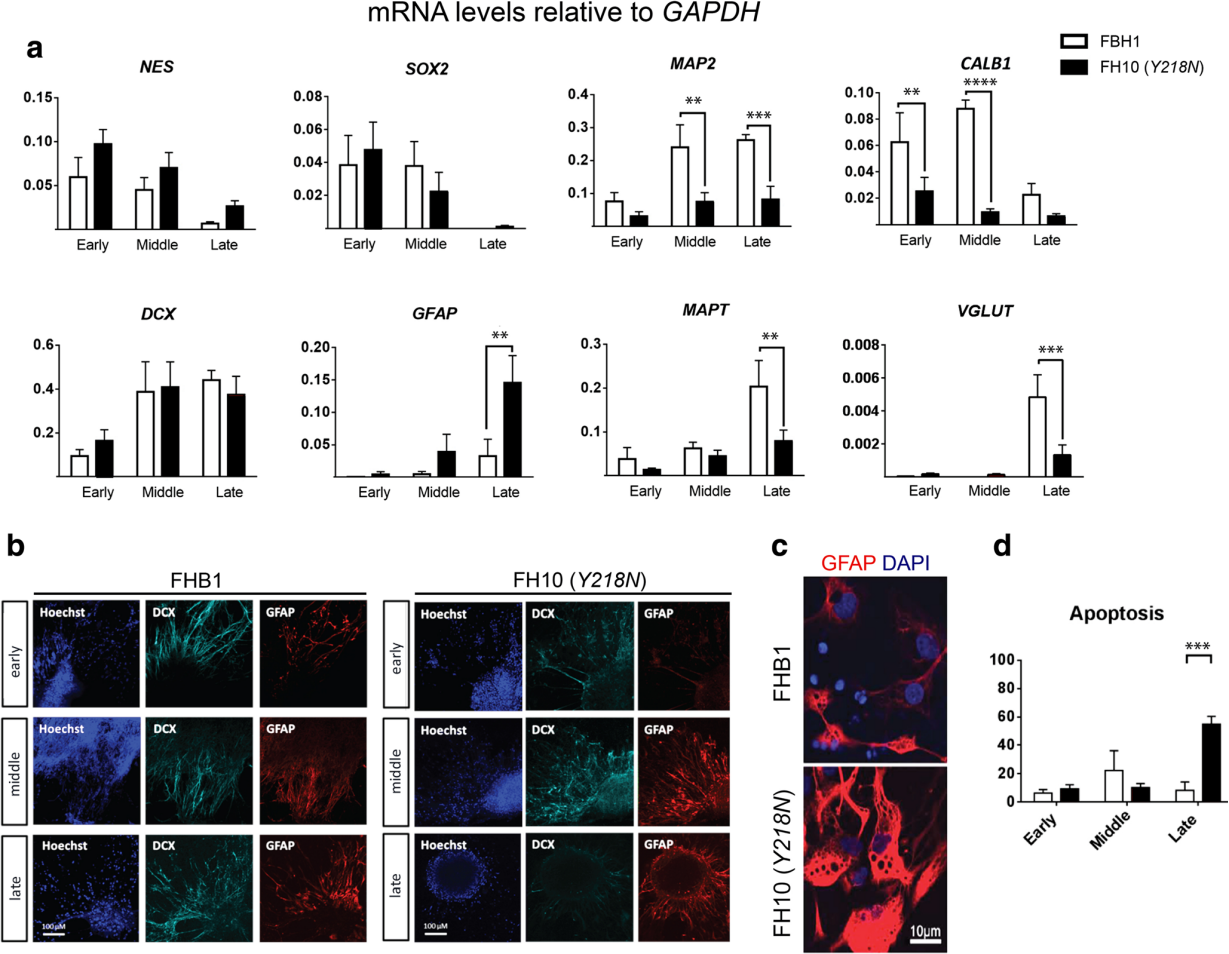
**Transcriptional profile of maturating iPS cell cultures. (a)** Quantitative RT-PCR transcriptional profile of control and mutant cell cultures at the three maturation stages. Bars represent the mean ± S.E.M. of 2–4 time points for each stage from at least 2 independent differentiations. Data are presented as mean ± standard error of the mean (S.E.M.). Differences between groups were considered statistically significant **** *P* < 0.001, *** *P* < 0.01 and ** *P* < 0.05. Bonferroni post hoc test. **(b**) Representative immunofluorescence microphotographs of PrP^C^, DCX and GFAP expression at the three differentiation stages. **(c**) Higher power image of GFAP positive cells at mid differentiation stage. **(d**). Quantification of apoptotic nuclei (% over total Hoechst). *** *P* < 0.01, Bonferroni post hoc test. Scale bars in b = 100 μm and c = 10 μm

We next examined the transcriptional profile of neural cultures determined by RT-qPCR from early to late culture stages (Fig. 3a) and observed significant differences between control and *Y218N* cultures, particularly at the late stage (>120 days). While there were no differences in early progenitor markers such as *NES* and *SOX2,* which showed a similar time-dependent downregulation in both genotypes, a few neuronal transcripts were lower in the *Y218N* cultures early on, like *MAP2* and *CALB*. At the late stage (>120 days), there was a robust increase in *GFAP* mRNA and a concomitant decrease in mature neuronal markers including *MAPT* and *VGLUT1* mRNAs in *Y218N* cultures compared to controls. Next, these mRNA changes were checked by immunohistochemistry and cell counts (Fig. 3b, d). Cell counts revealed that the relative percentage of DCX and GFAP expressing cells was not significantly different between control and *Y218N* cultures (data not shown). Thus, the transcriptional increase in *GFAP* expression most likely due to a greater expression in reactive astroglial cells (Fig. 3c). Indeed, detailed analysis of immunoreacted cells revealed high content of GFAP forming thick fascicles in hypertrophic reactive astroglial cells at the late stage of *Y218N* cultures (Fig. 3c). Lastly, nuclear staining analysis in differentiating cultures revealed increased chromatin condensation and apoptosis in the *Y218N* at the late stage (Fig. 3d).

### Increased tau Phosphorylation in *Y218N*-Derived Neurons in Vitro

**Fig. 4:**
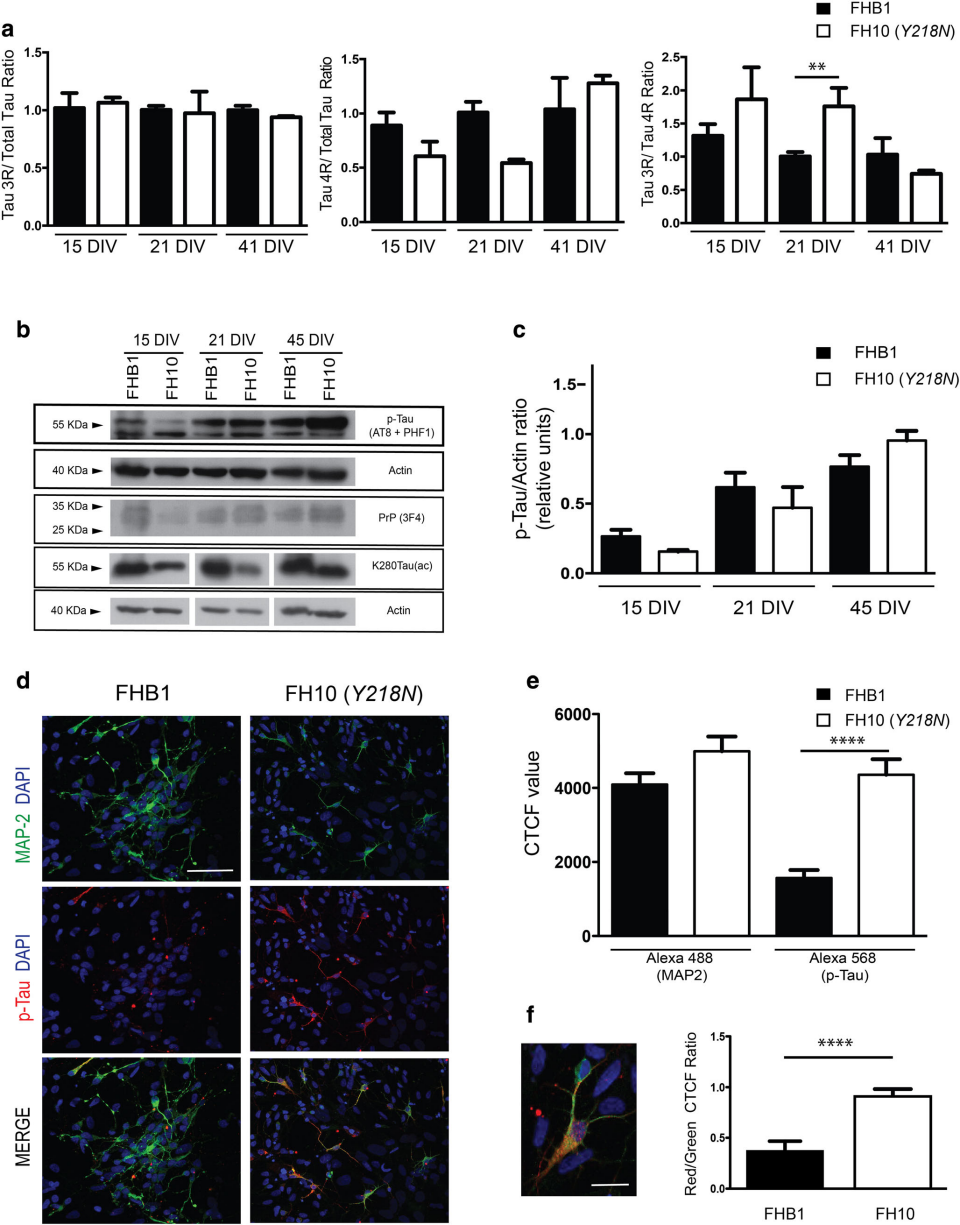
**Delayed** ***MAPT*** **maturation and increased p-Tau in** ***Y218N*** **-derived neurons. (a)** Histograms illustrating RT-qPCR results (mean ± S.E.M.) of Tau 3R/Total tau; Tau 4R/Total and Tau 3R/4R ratios in FHB1 and FH10 (Y218N) iPS cell cultures during differentiation at 15, 21 and 41 days in vitro. Asterisks in the right graph indicate *P* < 0.05, Bonferroni post hoc test; Mean Diff. -0.756; 95% confidence interval = −1.372 to - 0.1415). (**b)** Time course of p-Tau, PrP^C^ and K280Tau-(ac) expression in FHB1 and FH10 (*Y218N*) at 15, 21 and 45 DIV. Actin was used as control loading protein. **(c**) Graph of the densitometric values of p-Tau levels of (b). Plots show mean ± S.E.M. of three different experiments. Note the increase in p-Tau between *Y218N* and control cells. **(d)** High power photomicrographs illustrating MAP2 (green), p-Tau (red) in FHB1 and FH10 (*Y218N*) neural cultures. A high magnification of a labelled cell is showed in (**f**). **(e-f)** Quantification of CTCF values derived from experiments in (d). Plots show mean ± S.E.M. of four different experiments. Asterisks in (e) indicate statistical differences between groups and controls. **** *P* < 0.001; Mann-Whitney U test. Scale bars in d = 50 μm and f = 10 μm

As indicated above, clinical and histopathological examination of the GSS patient carrying the *Y218N PRNP* mutation displayed relevant neurofibrillary degeneration with p-Tau deposits in several brain regions [17]. Therefore, we explored putative changes in Tau expression and phosphorylation in differentiating cultures (Fig. 4, Supplementary Figs. 3 and 4). As indicated above, *MAP2* and *MAPT* mRNA levels decreased in *Y218N*-derived cultures compared to controls suggesting delayed neuronal differentiation (Fig. 3). This was corroborated analyzing the appearance of the two Tau-splicing forms (4R and 3R) in differentiated cultures (Fig. 4a). RT-qPCR analysis demonstrated a delayed appearance of the 4R Tau form compared to 3R Tau in *Y218N*-derived cultures (Fig. 4a). This was corroborated by the biochemical analysis of the acetylated form of Tau at lysine 280 (K280-(ac)) Tau during the differentiation (Fig. 4b, Supplementary Fig. 4). This acetylated form is associated with Tau 4R [42, 43]. K280-(ac) Tau levels were constant in control-derived cultures from 15 to 45 DIV. However, *Y218N* cultures showed increased levels of K280-(ac) Tau between 15 to 41 DIV, following changes of Tau 4R (Fig. 4b, Supplementary Fig. 4). In parallel, biochemical detection of p-Tau during differentiation demonstrated the increase in p-Tau (detected by AT8 and PHF1 antibodies) in *Y218N*-derived neurons compared to control without relevant changes in PrP^C^ protein levels (Fig. 4b,c, Supplementary Fig. 3).

Next, we developed a CTCF analysis of p-Tau in identified MAP2-positive neurons (Fig. 4d,e). First we counted the total number of MAP2 and p-Tau-positive neurons in *Y218N*- and control-derived cultures. As suggested above with RT-qPCR, the total number of MAP2 and p-Tau double-positive cells was lower in *Y218N*-derived cultures (Fig. 4d,e). In addition, the relative percentage of double-labeled p-Tau/MAP2 neurons was also higher in *Y218N*-derived neurons. Changes in p-Tau level in differentiated neurons were also corroborated by the CTCF analysis of p-Tau in double-labeled p-Tau/MAP2-immunoreactive neurons (Control: 1566 ± 214.4; *Y218N*: 4357 ± 422.1; mean ± S.E.M. *P* < 0.0001, Mann-Whitney *U* test). Indeed, the ratio of p-Tau/MAP2 fluorescence was higher in *Y218N*-derived neurons compared to control-derived neurons (Control: 0.27 ± 0.02; *Y218N*: 0.680 ± 0.02; mean ± S.E.M. *P* < 0.0001, Mann-Whitney *U* = 2628) (Fig. 4e,f). Unfortunately, electron microscopy analyses failed to identify neurofibrillary tangle formation in *Y218N* differentiated neurons (not shown). Similar biochemical observations were also made using the direct cortical differentiation protocol **(**Supplementary Fig. 5 **)**. In conclusion, cultures derived from *Y218N* mutant iPS cells recapitulated in vitro several pathological features of the GSS patient, such as reactive astrocytosis, cell death and Tau hyperphosphorylation.

### Impaired Mitochondria Movement in *Y218N*-Derived Neurons

**Fig. 5:**
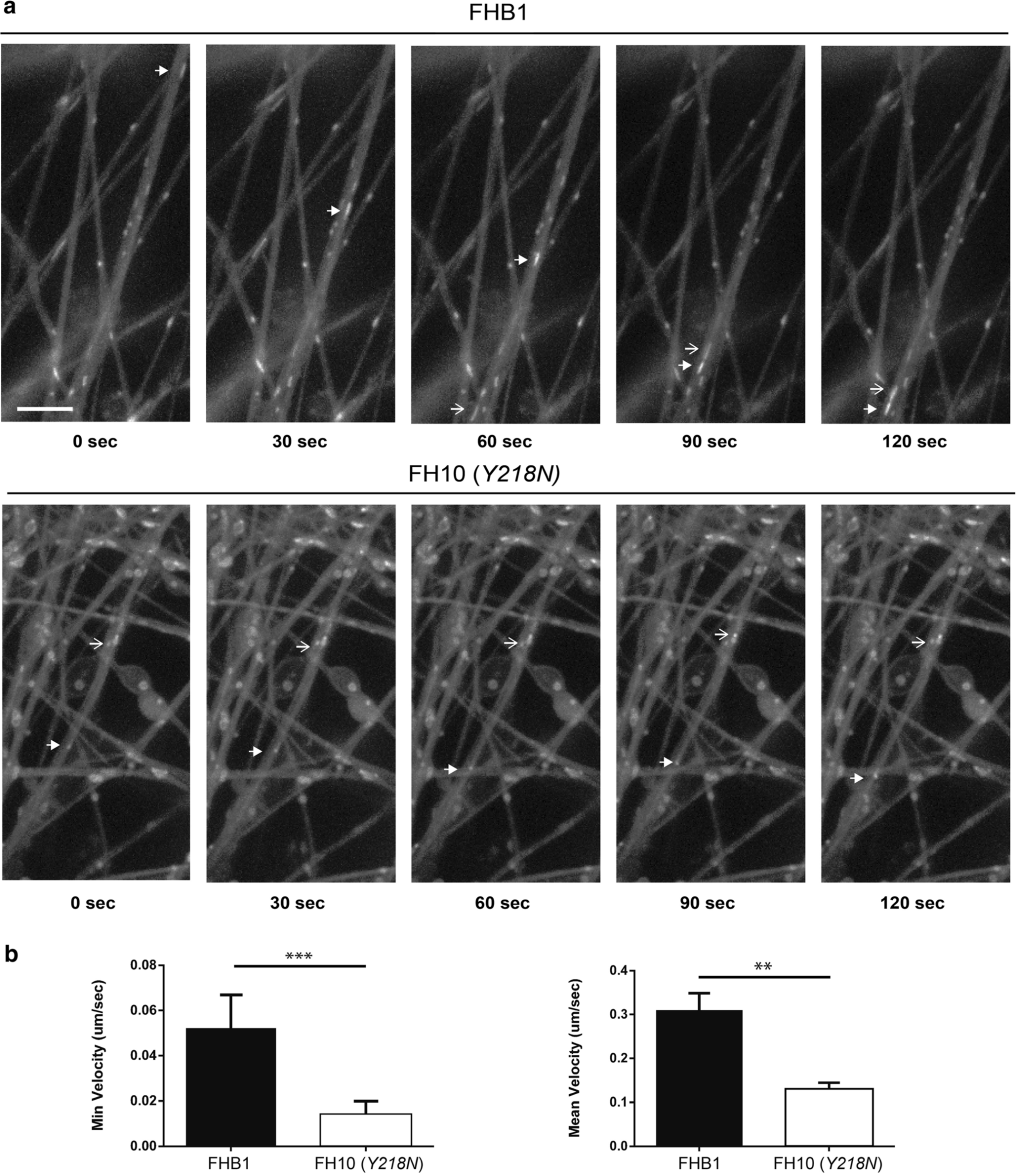
**FH10** ***(Y218N)*** **cultures showed impaired mitochondria displacement. (a)** Time-lapse fluorescence photomicrographs illustrating mitochondria movement in FHB1- (upper panels) and FH10 (*Y218N*)- (lower panels) derived neurons. The movement of two mitochondria (arrow and open arrow in (a) can be seen in the time lapse panels. **(b)** Plots illustrating the Minimum and Mean velocity values of tracked mitochondria in both types of cultures (see Methods for details). Notice the strong decreases in velocity in FH10 (*Y218N*)-derived cultures. Plots show mean ± S.E.M. of three different differentiation experiments. ***P < 0.01, ** *P* < 0.05. Mann Whitney *U* test. Scale bar: a = 2.5 μm

The effects of Tau hyperphosphorylation in several epitopes on mitochondria movement have been demonstrated in Alzheimer’s disease [44, 28]. We checked the minimum and the mean velocity of identified mitochondria (Fig. 5). Results showed a decrease in both measurements in *Y218N* cultures compared with control (0.052 ± 0.014 (control, *n* = 111) vs 0.014 ± 0.006 (*Y218N*; *n* = 105); mean ± S.E.M., Min. velocity in μm/s; *P* = 0.0004; Mann-Whitney U value =4688. 0.310 ± 0.038 (control, *n* = 111) vs 0.1302 ± 0.014 (*Y218N*; *n* = 105); Mean ± S.E.M. Mean velocity in μm/s; *P* = 0.0484; Mann-Whitney U value =4921) (Fig. 5b).

### Infectivity Assays

**Fig. 6:**
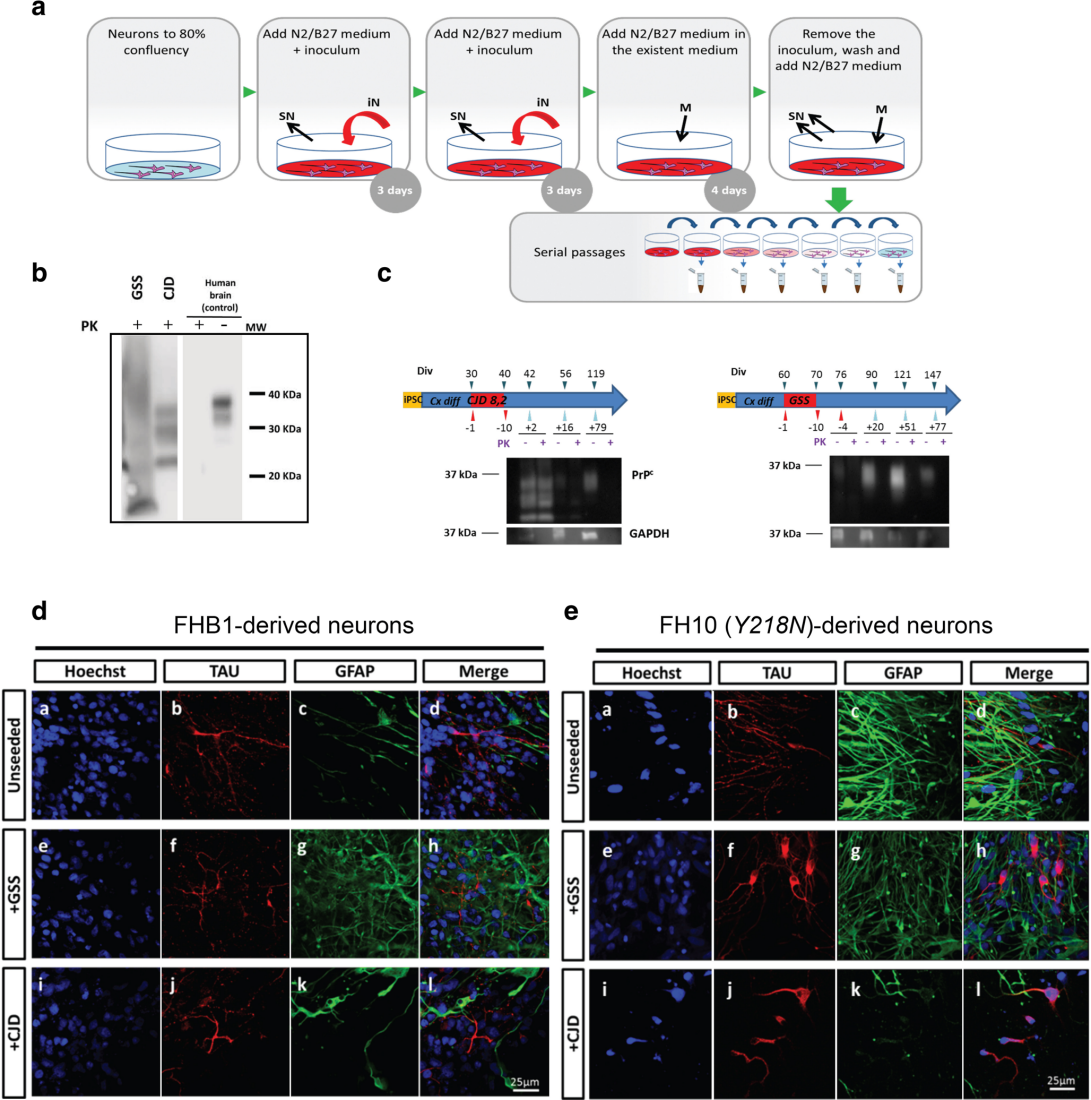
**Infectivity assay with brain inoculates**. **(a**) Schematic representation of the inoculation protocol: infective brain homogenates were added at day 0 and day 3 and removed at day 10; cells were subsequently passaged several times to remove the inocula. (**b**) Inocula from the sources (10% of brain homogenates, see Methods for details) were processed to show PK-resistant PrP signal. *GSS*: human brain diagnosed of *Y218N*. *CJD*: human brain diagnosed of a sporadic CJD MM1. CJD samples were digested with 10–50 μg/ml of proteinase K (PK) and subjected to a standard biochemical analysis. GSS sample was treated as an atypical prion sample (see Methods). The samples were analyzed using the monoclonal antibody 3F4. MW: Molecular marker. **(c**) Representative examples of Western blot detection of PK-resistant PrP forms following inoculation with CJD and GSS brain samples. Note that PK-resistant PrP was only detected (when present) for the first 2 weeks after the infection. **(d**) Morphological analyses 2 months later revealed little effect of these inoculates in control neurons while mutant *Y218N* cultures (**e**) showed fewer neurons with marked cytoplasmic redistribution of Tau signal (**b**,** f,** **j**) and enhanced immunoreactivity for GFAP. Scale bars: 25 μm

In order to examine susceptibility to prion infection, *Y218N*- and control-differentiated cultures were exposed for 10 days to brain homogenates prepared from a sporadic CJD and from a GSS *Y218N* brain (Fig. 6a,b). Western blot analyses of the cultures showed the presence of PK-resistant PrP forms but only up to 2 weeks after removal of the inocula, indicating lack of prion infectivity (and propagation) in these conditions (Fig. 6c). A similar signal was observed in cultures infected with 263 K, a hamster scrapie prion strain which does not propagate to human cells (data not shown). Despite the absence of PK-resistant PrP in the cultures after two weeks, we observed some phenotypic changes that were more prominent in mutant neurons. In particular, we found a prominent redistribution of Tau signal with enhanced localization in the soma and proximal neurites in *Y218N* neurons exposed to either *GSS* or *CJD* inoculates was found (Fig. 6d,e).

## Discussion

In the present study we have developed, for the first time, an iPS cell model of a familial human prionopathy. The donor GSS patient carrying the *Y218N PRNP* mutation showed relevant gliosis, cell death, massive deposits of PrP and neurofibrillary degeneration in different brain regions [17]. Surprisingly, the presence of the *Y218N* mutation in other family member belies atypical parkinsonism phenotype instead of neurofibrillary degeneration [32]. Indeed, in parallel with the classical signs of GSS-associated degeneration, other clinical presentations, such as Alzheimer’s-type, frontotemporal-like dementia, parkinsonism, and atypical psychiatric disorders, have all been reported (e.g., [45]). These different clinical manifestations have also been found in family members with the same *PRNP* mutation, attributed to the distinct abnormal isoforms of prion protein and polymorphisms at codon 129 [46, 47]. In fact, in this case the two GSS patients differ at the codon 129 polymorphism (129MV [32] and 129VV in [17]), which may contribute to clinical differences between cases.

We used two different well-characterized procedures to differentiate the iPS cells into neurons [34, 26]. The first was directed to obtaining forebrain cortical neurons [34] and the second one was directed to maintaining regulated developmental steps during neural development [26]. With both protocols we obtained similar results being able to determine that *Y218N*-derived cultures showed relevant GFAP reactivity, cell death, neuronal Tau redistribution, elevated p-Tau levels and changes in mitochondrial trafficking. Despite being unable to reproduce the spontaneous generation of PK-resistant forms or enabling prion propagation after inoculation in *Y218N*-iPS cell derived cultures, the differentiated neural cells recapitulated most of the pathological features observed in the GSS patient’s brain. In this way, these *Y218N*-derived cultures could be used as an in vitro platform for neurodegenerative studies in familial prionopathies with the aim of characterizing the role of particular *PRNP* mutations in comorbid taupathies and cell death.

Unfortunately, *Y218N* cells did not generate PrP spontaneously and they were unable to propagate human prions (CJD and *Y218N* GSS prions) in vitro. This was disappointing but certainly not fully unexpected given that it has never been possible to propagate infectivity in primary neurons with human prions. Furthermore there are no studies of *Y218N* PrP propagation in vivo in contrast to other human mutations: i) *P102L* GSS human prion in *P101L* mice [48], ii) *A117V* GSS-derived human prion inoculum in *AV117 PRNP mice* [49] and iii) *P102L*, *A117V* or *F198S PRNP* mutations in bank voles [50].

Concerning the absence of endogenous prion generation in *Y218N*-derived neurons we might hypothesize, in a simplistic manner, that current in vitro times are not long enough for the endogenous generation of human PrP^res^, considering the clinical onset and evolution of the GSS patient. However, we believe that the current scenario is not as simple, and that other, as yet unknown factors with key functions in protein misfolding and propagation may be absent from our cultures. In vitro prion propagation (of mostly mouse adapted strains) has been developed in neural and non-neural cell lines [51], primary neuronal cultures [52], cerebellar organotypic slices [53], and, with some controversy, in neurospheres (e.g., [54]). However, human prions have not been propagated in neuronal cultures to date. In fact, a single study of Ladogama et al. reported the transmission of human prions but using neuroblastoma cells [55]. In addition, the propagation of human strains was more successful when prions had been previously adapted to mice (e.g., M1000 [56]). Although endogenous expression levels could be relevant, we cannot rule out the participation of other non-neuronal cells (microglial cells) and inflammatory processes in protein misfolding and propagation [57] which did not fully develop in our iPS cell cultures, in contrast to other 3D organotypic approaches that could be assayed in future experiments [58].

In our study, the differentiation of *Y218N*-derived iPS cells was protracted. It is well known that appropriate temporal and transcriptional levels of *PRNP* are required for the correct differentiation of human embryonic stem cells [59] as well as other neural stem cells in vivo [60] and in vitro [61]. In fact, early attempts to ablate *PRNP* in mice using constitutive promoters and large *PRNP* mutations were not viable because *PRNP* expression starts around E7.5 in neural tissue [62]. Indeed, PrP^C^ is involved in several neural and non-neural developmental functions and its absence either delays or interferes with cell proliferation and maturation [63–65]. Although a clear explanation of the physiological impact of the *Y218N* mutation in these processes remains elusive, the mutation might induces aberrant folding of the protein [66], which may impair neuronal differentiation. In fact, in our experiments, mutant neurons showed decreased mRNA levels of *CALB*, *VGLUT1* and *MAP2* compared with control cells. In addition, *Y218N*-derived neurons showed very low numbers of Ca^2+^ transients analyzed by Fluo4-AM (data not shown).

The deposition of hyperphosphorylated forms of Tau (p-Tau) has been described in familial and sporadic forms of prion diseases and in the brains of patients with variant CJD. Elevated levels of Tau (p-Tau and total Tau) have also been reported in the cerebrospinal fluid (CSF) of patients with sporadic CJD [67]. In addition, rodents infected with BSE [68], 263 K [69] and human CJD [70] derived inocula also showed elevated levels of p-Tau. Indeed, neurons in encephalopathy-affected brain regions displaying PrP aggregates showed relevant Tau redistribution with increased perinuclear location. This perinuclear Tau reorganization was observed in *CJD*- and *GSS*-treated *Y218N*-derived neurons in this study. In this matter, it is well known that aggregated prion peptides [71], as well as infectious prions [70], may modulate microtubule dynamics and stability, which may also in turn implicate Tau distribution. The increased presence and neuronal redistribution of Tau likely has a direct effect on the neuropathological process triggered by prion presence, because PrP^C^ binding to Tau is probably disrupted by the mutations (at least for P102L *PRNP* [18]). In fact, if we consider that *Y218N* might alter natural PrP^C^ functions associated with Tau, the cellular responses mediated by sCJD and GSS prions might be exacerbated in the presence of the *Y218N PRNP* mutation.

In conclusion, we report here the use of iPS cell-derived neurons to investigate the putative roles of the *Y218N PRNP* mutation in neural differentiation, Tau phosphorylation and cell death. This approach provides a powerful in vitro system for functional analysis of pathways regulating *PRNP* function in human cortical neurons, cellular mechanisms regulating tau phosphorylation in these models and for the identification and testing of candidate disease-modifying compounds.

## Electronic supplementary material

Supplementary Table 1 (DOCX 14 kb)

Supplementary Table 2 (DOCX 15.3 kb)

Supplementary Table 3 (DOCX 13.6 kb)

Supplementary Fig. 1 Full size uncropped blots corresponding to Fig. 2g, IPS (asterisk in a) and SNM (asterisk in b). (JPEG 4.13 mb)

Supplementary Fig. 2 High ECL exposure (15 min) showing the determination of PK-resistant PrP^C^ levels illustrated in Fig. 2h. Uncropped film. (JPEG 621 kb)

Supplementary Fig. 3 Full size uncropped blots corresponding to Fig. 4b (upper panels). Red arrows point to the proteins of interest. Data from 15, 21 and 45 DIV are shown in Fig. 4b. (JPEG 4.06 mb)

Supplementary Fig. 4 Full size uncropped blots corresponding to Fig. 4b (lower panels). (JPEG 1.15 mb)

Supplementary Fig. 5 Determination of p-Tau (AT8 antibody) levels in FHB1 and FH10 (*Y218N*)-derived cultures at several DIV of differentiation (procedure 1, see Methods). p-Tau probed membranes were immunoblotted using antibodies against GADPH for standardization. (JPEG 488 kb)
