# Supplementary material for: iPS Cell Cultures from a Gerstmann-Sträussler-Scheinker Patient with the Y218N PRNP Mutation Recapitulate tau Pathology
Source: Mol Neurobiol. 2017 May 2;55(4):3033–48. doi: 10.1007/s12035-017-0506-6 (PMC5842509; doi:10.1007/s12035-017-0506-6)
Supplement: Supplementary file 1 — (DOCX 14 kb) [file 12035_2017_506_MOESM1_ESM.docx]

**Supplementary Table 1.** Primers used in Figure 1 and the analyses used for and their sequencing.

qPCR Total OCT4 Forward 5’-GGAGGAAGCTGACAACAATGAAA-3’

qPCR Total OCT4 Reverse 5’-GGCCTGCACGAGGGTTT-3’

qPCR Total SOX2 Forward 5’-TGCGAGCGCTGCACAT-3’

qPCR Total SO2 Reverse 5’-TCATGAGCGTCTTGGTTTTCC-3’

qPCR Total KLF4 Forward 5’-CGAACCCACACAGGTGAGAA-3’

qPCR Total KLF4 Reverse 5’-GAGCGGGCGAATTTCCAT-3’

qPCR Total c-MYC Forward 5’-AGGGTCAAGTTGGACAGTGTCA-3’

qPCR Total c-MYC Reverse 5’-TGGTGCATTTTCGGTTGTTG-3’

qPCR Trans OCT4 Forward 5’-TGGACTACAAGGACGACGATGA-3’

qPCR Trans OCT4 Reverse 5’-CAGGTGTCCCGCCATGA-3’

qPCR Trans SOX2 Forward 5’-GCTCGAGGTTAACGAATTCATGT-3’

qPCR Trans SOX2 Reverse 5’-GCCCGGCGGCTTCA-3’

qPCR Trans KLF4 Forward 5’-TGGACTACAAGGACGACGATGA-3’

qPCR Trans KLF4 Reverse 5’-CGTCGCTGACAGCCATGA-3’

qPCR Trans c-MYC Forward 5’-TGGACTACAAGGACGACGATGA-3’

qPCR Trans c-MYC Reverse 5’-GTTCCTGTTGGTGAAGCTAACGT-3’

qPCR NANOG Forward 5’-ACAACTGGCCGAAGAATAGCA-3’

qPCR NANOG Reverse 5’-GGTTCCCAGTCGGGTTCAC-3’

qPCR CRIPTO Forward 5’-CGGAACTGTGAGCACGATGT-3’

qPCR CRIPTO Reverse 5’-GGGCAGCCAGGTGTCATG-3’

qPCR REX1 Forward 5’-CCTGCAGGCGGAAATAGAAC-3’

qPCR REX1 Reverse 5’-GCACACATAGCCATCACATAAGG-3’

qPCR GAPDH Forward 5’-GCACCGTCAAGGCTGAGAAC-3’

qPCR GAPDH Reverse 5’-AGGGATCTCGCTCCTGGAA-3’

Bisulfite seq OCT4 Forward 5’-GGATGTTATTAAGATGAAGATAGTTGG-3’

Bisulfite seq OCT4 Reverse 5’-CCTAAACTCCCCTTCAAAATCTATT-3’

Bisulfite seq NANOG Forward 5’-AGAGATAGGAGGGTAAGTTTTTTTT-3’

Bisulfite seq NANOG Reverse 5’-ACTCCCACACAAACTAACTTTTATTC-3’

Integration KLF4 Forward 5’-AATTACCCATCCTTCCTGCC-3’

Integration KLF4 Reverse 5’-TTAAAAATGCCTCTTCATGTGTA-3’

Integration OCT4 Forward 5’-TAAGCTTCCAAGGCCCTCC-3’

Integration OCT4 Reverse 5’-CTCCTCCGGGTTTTGCTCC-3’

Integration SOX2 Forward 5’-AGTACAACTCCATGACCAGC-3’

Integration SOX2 Reverse 5’-TCACATGTGTGAGAGGGGC-3’

Integration c-MYC Forward 5’-TCCACTCGGAAGGACTATCC-3’

Integration c-MYC Reverse 5’-TTACGCACAAGAGTTCCGTAG-3’

Sequentiation Mut218N Forward 5’- GCCAAAAACCAACATGAAGC -3’

Sequentiation Mut218N Reverse 5’- CATGCTCGATCCTCTCTGG -3’
