# Supplementary material for: iPS Cell Cultures from a Gerstmann-Sträussler-Scheinker Patient with the Y218N PRNP Mutation Recapitulate tau Pathology
Source: Mol Neurobiol. 2017 May 2;55(4):3033–48. doi: 10.1007/s12035-017-0506-6 (PMC5842509; doi:10.1007/s12035-017-0506-6)
Supplement: Supplementary file 2 — (DOCX 15.3 kb) [file 12035_2017_506_MOESM2_ESM.docx]

**Supplementary Table 2.** Antibodies used in the present study.

| **Antibody** | **Cat. No.** | **Origin** | **Supplier** | **Dilution** |
| --- | --- | --- | --- | --- |
| α-Fetoprotein | A0008 | Rabbit | Dako | 1:400 |
| Actin | 1A4 | Mouse | Sigma | 1:5000 |
| AT8 | MN1020 | Mouse | Invitrogen | 1:1000 |
| DCX | Goat | Goat | Santa Cruz | 1:100 |
| FoxA2 | AF2400 | Goat | R&D Biosystems | 1:100 |
| GADPH | AB9485 | Rabbit | Abcam | 1:300 |
| GATA4 | sc-9053 | Rabbit | Santa cruz | 1:50 |
| GFAP | Z0034 | Rabbit | Dako | 1:500 |
| K280Tau-(ac) | AS-56077 | Rabbit | Anaspec | 1:500 |
| Ki67 | AB9260 | Rabbit | Millipore | 1:300 |
| MAP2 | M1406 | Rabbit | Sigma | 1:100 |
| Nanog | AF1997 | Goat | Everest Biotech | 1:100 |
| Nestin | AB5922 | Rabbit | Chemicon | 1:250 |
| NeuN | MAB377 | Mouse | Chemicon | 1:250 |
| Oct4 | sc-5279 | Mouse | Santa Cruz | 1:100 |
| Pax6 | PRB-278P | Rabbit | Covance | 1:100 |
| PFH1 | gift | Mouse | Prof. D. Harris | 1:150 |
| PrP (3F4) | MAB1562 | Mouse | Prionics | 1:500 |
| Sox2 | PA1-16968 | Rabbit | Chemicon | 1:500 |
| SSEA3 | MC-631 | Rat | Hybridoma Bank Iowa | 1:2 |
| SSEA4 | MC-813-70 | Mouse | Hybridoma Bank Iowa | 1:2 |
| SMA | A5228 | Mouse | Sigma | 1:400 |
| Tau5 | MAB361 | Mouse | Millipore | 1:100 |
| TRA-1-81 | MAB4381 | Mouse | Millipore | 1:200 |
| TUJ1 | MMS-435P | Mouse | Covance | 1:500 |
