# Supplementary material for: iPS Cell Cultures from a Gerstmann-Sträussler-Scheinker Patient with the Y218N PRNP Mutation Recapitulate tau Pathology
Source: Mol Neurobiol. 2017 May 2;55(4):3033–48. doi: 10.1007/s12035-017-0506-6 (PMC5842509; doi:10.1007/s12035-017-0506-6)
Supplement: Supplementary file 3 — (DOCX 13.6 kb) [file 12035_2017_506_MOESM3_ESM.docx]

**Supplementary Table 3.** Primers used in the iPS cell differentiation (Fig. 3 and 4) and their sequencing.

qPCR Total CALB1 Forward 5’- GACGGAAGTGGTTACCTGGA-3’

qPCR Total CALB1 Reverse 5’- TGCCCATACTGATCCACAAA -3’

qPCR Total NES Forward 5’- ACAGCCATAGAGGGCAAAGTGGTA -3’

qPCR Total NES Reverse 5’- AAGGAACCTGGGAGTCCTGGATTT -3’

qPCR Total SOX2 Forward 5’- GGGAAATGGGAGGGGTGCAAAAGAGG -3’

qPCR Total SOX2 Reverse 5’- TTGCGTGAGTGTGGATGGGGATTGGTG -3’

qPCR Total DCX Forward 5’- CATCCCCAACACCTCAGAAG -3’

qPCR Total DCX Reverse 5’- GGAGGTTCCGTTTGCTGA -3’

qPCR Total MAP2 Forward 5’- CCGTGTGGACCATGGGGCTG -3’

qPCR Total MAP2 Reverse 5’- GTCGTCGGGGTGATGCCACG -3’

qPCR Total MAPT Forward 5’- GATTGGGTCCCTGGACAATA -3’

qPCR Total MAPT Reverse 5’- GTGGTCTGTCTTGGCTTTGG -3’

qPCR Total GFAP Forward 5’- TCTCTCGGAGTATCTGGGAACTG -3’

qPCR Total GFAP Reverse 5’- TTCCCTTTCCTGTCTGAGTCTCA -3’

qPCR Total VGLUT1 Forward 5’- GAAGGTGAAGGTCGGAGT -3’

qPCR Total VGLUT1 Reverse 5’- GAAGATGGTGATGGGATTTC -3’

qPCR Total GAPDH Forward 5’- CACGTGGTGGTGCAGAAA 3’

qPCR Total GAPDH Reverse 5’- CGTGTATGAGGCCGACAGT -3’

qPCR Total 3R MAPT Forward 5’- GTCAGGTCGAAGATTGGCTCTACT -3’

qPCR Total 3R MAPT Reverse 5’- GCTTGTAGACTATTTGCACCTTGC -3’

qPCR Total 4R MAPT Forward 5’- TGTCAGGTCGAAGATTGGCTC -3’

qPCR Total 4R MAPT Reverse 5’- CTTATTAATTATCTGCACCTTGCCAC -3’
