# Supplementary figures and images for: iPS Cell Cultures from a Gerstmann-Sträussler-Scheinker Patient with the Y218N PRNP Mutation Recapitulate tau Pathology
Source: Mol Neurobiol. 2017 May 2;55(4):3033–48. doi: 10.1007/s12035-017-0506-6 (PMC5842509; doi:10.1007/s12035-017-0506-6)

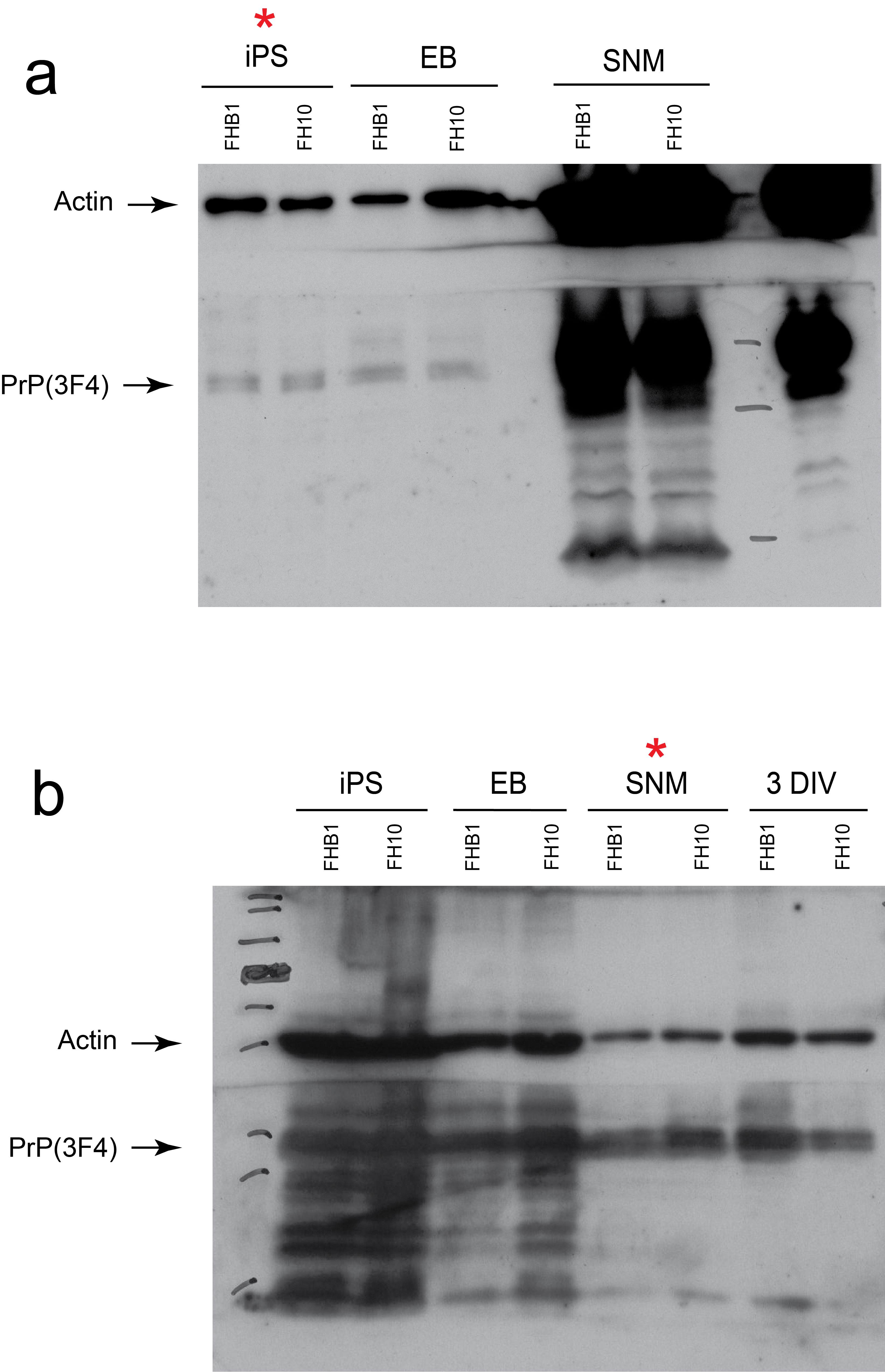

Supplement: Supplementary file 4 — Full size uncropped blots corresponding to Fig. 2g, IPS (asterisk in a) and SNM (asterisk in b). (JPEG 4.13 mb) [file 12035_2017_506_MOESM4_ESM.jpg]

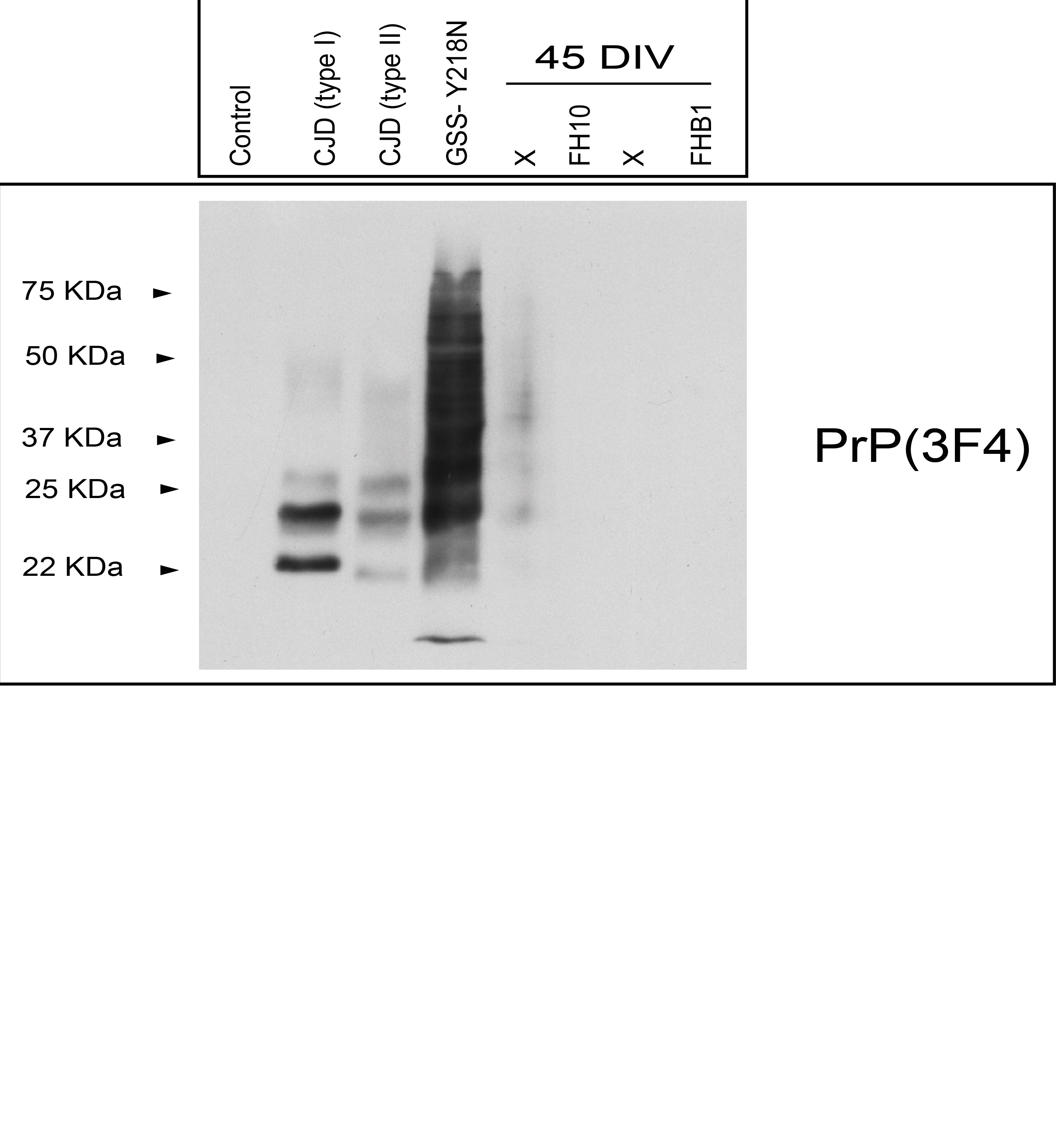

Supplement: Supplementary file 5 — High ECL exposure (15 min) showing the determination of PK-resistant PrPC levels illustrated in Fig. 2h. Uncropped film. (JPEG 621 kb) [file 12035_2017_506_MOESM5_ESM.jpg]

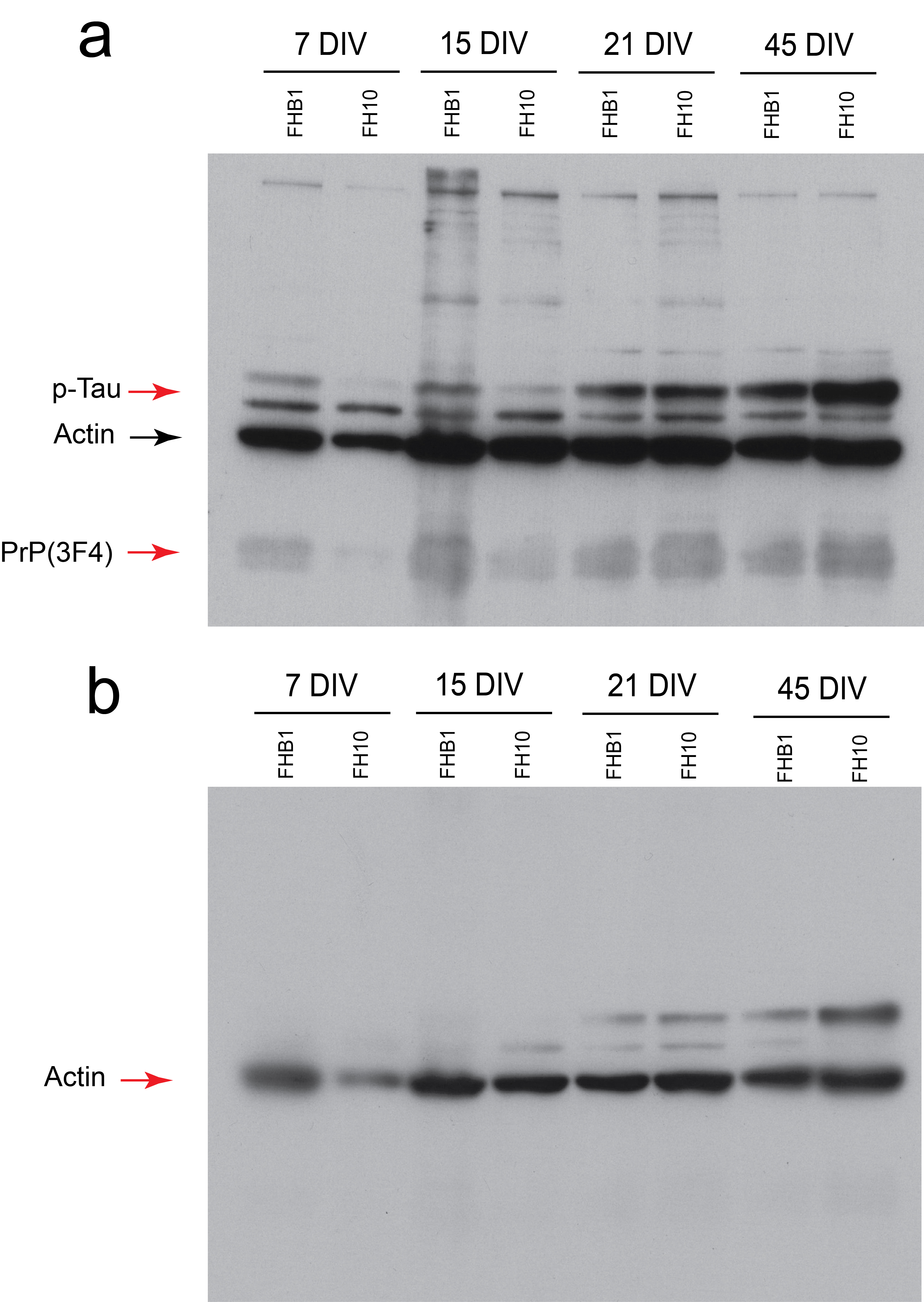

Supplement: Supplementary file 6 — Full size uncropped blots corresponding to Fig. 4b (upper panels). Red arrows point to the proteins of interest. Data from 15, 21 and 45 DIV are shown in Fig. 4b. (JPEG 4.06 mb) [file 12035_2017_506_MOESM6_ESM.jpg]

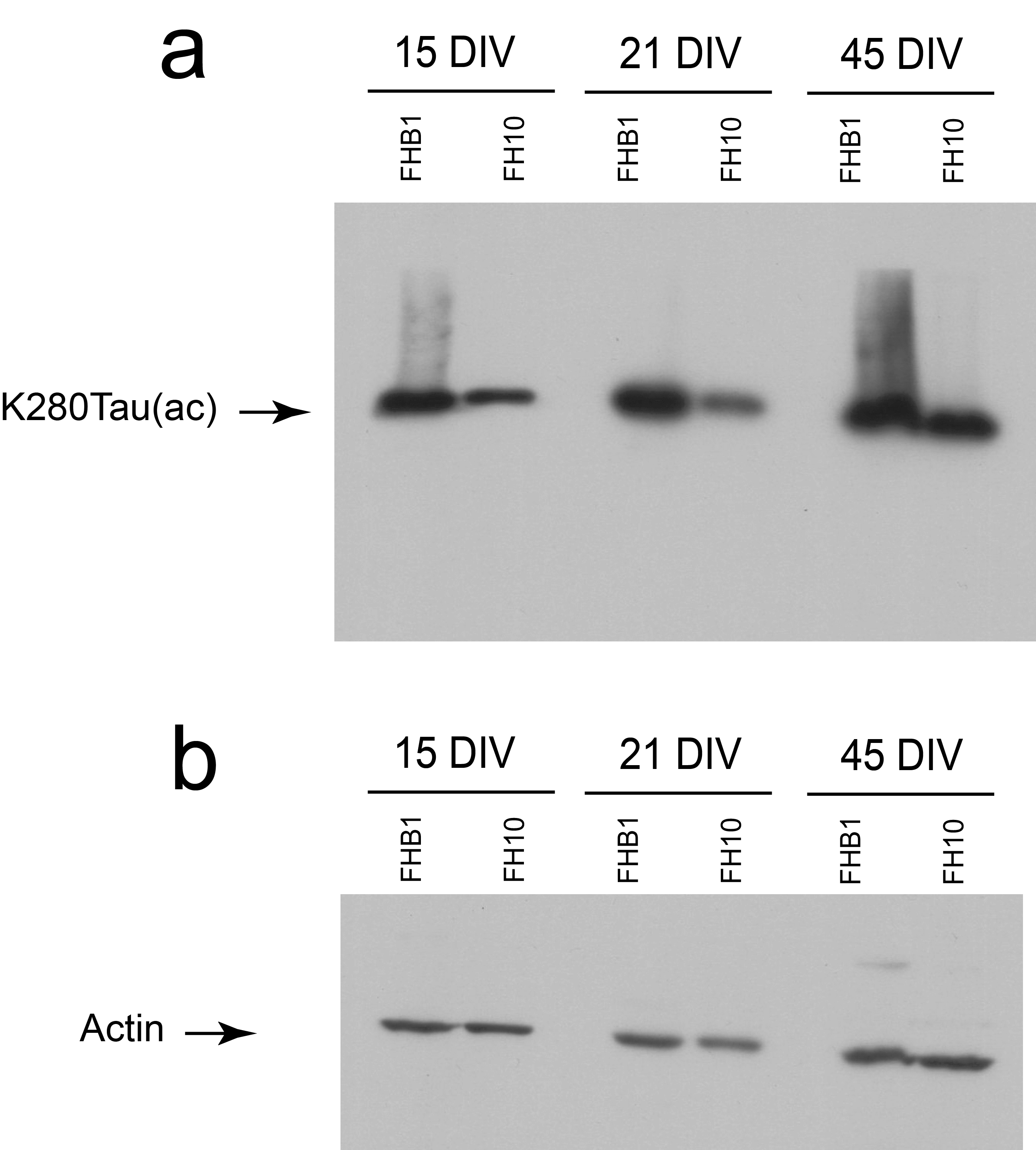

Supplement: Supplementary file 7 — Full size uncropped blots corresponding to Fig. 4b (lower panels). (JPEG 1.15 mb) [file 12035_2017_506_MOESM7_ESM.jpg]

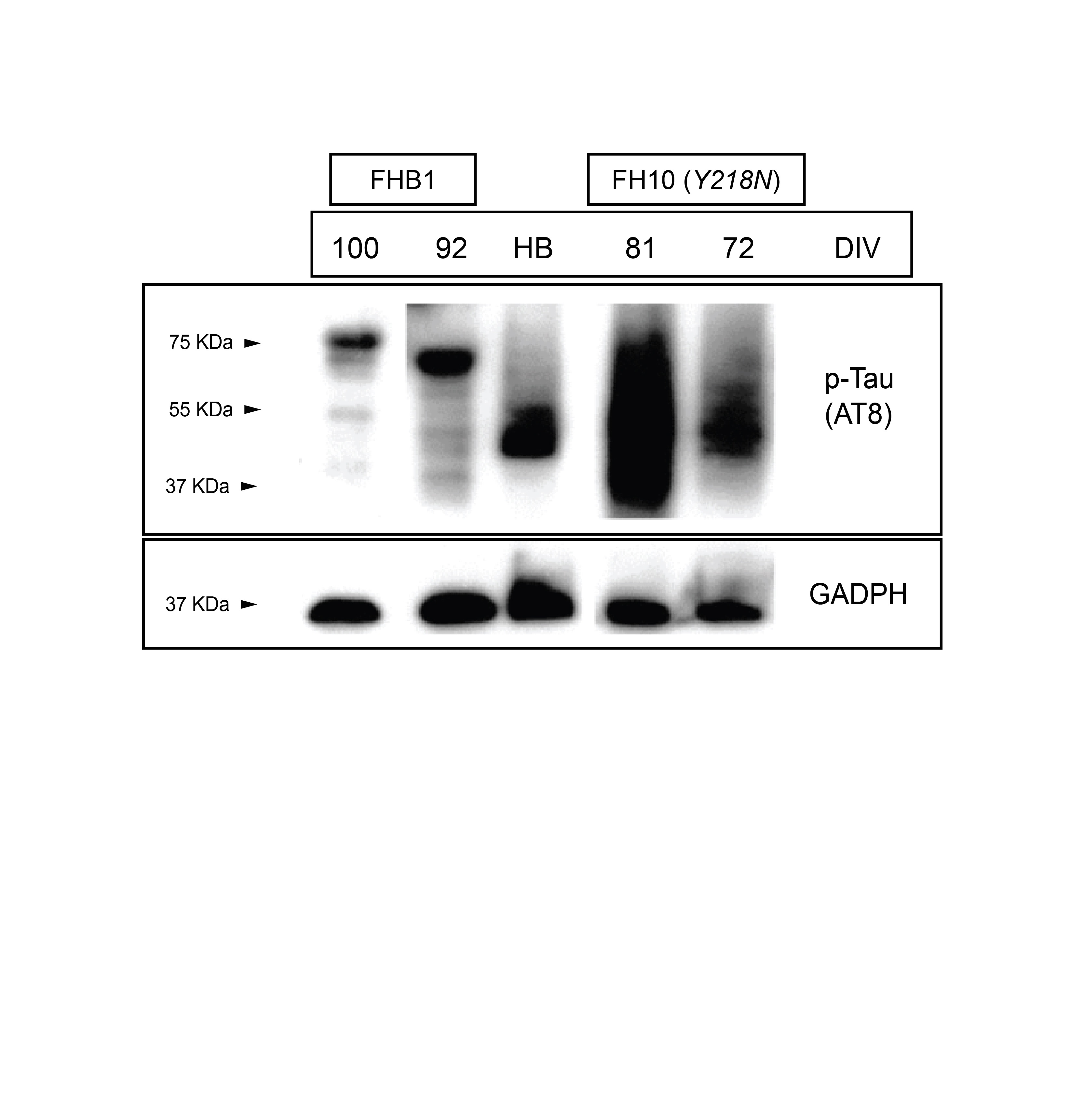

Supplement: Supplementary file 8 — Determination of p-Tau (AT8 antibody) levels in FHB1 and FH10 (Y218N)-derived cultures at several DIV of differentiation (procedure 1, see Methods). p-Tau probed membranes were immunoblotted using antibodies against GADPH for standardization. (JPEG 488 kb) [file 12035_2017_506_MOESM8_ESM.jpg]
